# Regulation of BolA abundance mediates morphogenesis in *Fremyella diplosiphon*

**DOI:** 10.3389/fmicb.2015.01215

**Published:** 2015-11-05

**Authors:** Shailendra P. Singh, Beronda L. Montgomery

**Affiliations:** ^1^MSU-DOE Plant Research Laboratory, Michigan State University, East LansingMI, USA; ^2^Department of Biochemistry and Molecular Biology, Michigan State University, East LansingMI, USA

**Keywords:** BolA, cellular morphology, complementary chromatic acclimation (CCA), cyanobacteria, light signaling, photomorphogenesis

## Abstract

Filamentous cyanobacterium *Fremyella diplosiphon* is known to alter its pigmentation and morphology during complementary chromatic acclimation (CCA) to efficiently harvest available radiant energy for photosynthesis. *F. diplosiphon* cells are rectangular and filaments are longer under green light (GL), whereas smaller, spherical cells and short filaments are prevalent under red light (RL). Light regulation of *bolA* morphogene expression is correlated with photoregulation of cellular morphology in *F. diplosiphon*. Here, we investigate a role for quantitative regulation of cellular BolA protein levels in morphology determination. Overexpression of *bolA* in WT was associated with induction of RL-characteristic spherical morphology even when cultures were grown under GL. Overexpression of *bolA* in a Δ*rcaE* background, which lacks cyanobacteriochrome photosensor RcaE and accumulates lower levels of BolA than WT, partially reverted the cellular morphology of the strain to a WT-like state. Overexpression of BolA in WT and Δ*rcaE* backgrounds was associated with decreased cellular reactive oxygen species (ROS) levels and an increase in filament length under both GL and RL. Morphological defects and high ROS levels commonly observed in Δ*rcaE* could, thus, be in part due to low accumulation of BolA. Together, these findings support an emerging model for RcaE-dependent photoregulation of BolA in controlling the cellular morphology of *F. diplosiphon* during CCA.

## Introduction

Cyanobacteria are important components of aquatic ecosystems where they perform ecologically important functions of photosynthesis and nitrogen fixation. In aquatic systems, both quality and quantity of light are known to differ at different depth levels (Postius et al., 2001), which can limit photosynthesis and nitrogen fixation. However, cyanobacteria have evolved peripheral light-harvesting structures called phycobilisomes (PBSs), which are attached to thylakoid membranes at the site of the photosystems, to efficiently harvest available photons (Shi et al., 2011; Gutu and Kehoe, 2012; Singh et al., 2015). PBSs are primarily composed of pigmented phycobiliproteins (PBPs), e.g., phycoerythrin (PE; λ_max_ ∼565 nm), phycocyanin (PC; λ_max_ ∼620 nm) and allophycocyanin (AP; λ_max_ ∼650 nm), and largely non-pigmented linker proteins (Bogorad, 1975; Kehoe and Gutu, 2006; Singh et al., 2015). Some cyanobacteria have developed an ability to change the pigment and/or protein composition of rods of their PBSs in response to changes in the quality or predominant wavelength(s) of light in the environment, which allows cyanobacteria to sus tain photosynthesis and nitrogen fixation in prevailing light conditions (Campbell, 1996; Postius et al., 2001). The rods are primarily composed of PE under green light (GL), whereas PC constitute the rods of PBSs under red light (RL) in *Fremyella diplosiphon* (Gutu and Kehoe, 2012). This ecologically important phenomenon is known as complementary chromatic acclimation (CCA) and has been well characterized in the filamentous, freshwater cyanobacterium *F. diplosiphon* (Bennett and Bogorad, 1973; Kehoe and Gutu, 2006; Kehoe, 2010). CCA permits *F. diplosiphon*, also known as *Tolypothrix* sp. PCC 7601 (Yerrapragada et al., 2015), to tune photosynthesis to different light qualities by tuning the pigment composition of rods of PBSs (Campbell, 1996; Kehoe and Gutu, 2006; Gutu and Kehoe, 2012).

Complementary chromatic acclimation is controlled by a phytochrome-related cyanobacteriochrome RcaE which is known to sense the change in quality of light and promote GL- or RL-dependent abundance of PE or PC, respectively, in PBS rods (Kehoe and Grossman, 1996; Terauchi et al., 2004). RcaE possesses light-regulated kinase activity and mediates light-dependent activation or repression of transcription of PC or PE biosynthetic genes via its cognate response regulators (RR) RcaF and DNA-binding RcaC (Li et al., 2008; Bezy and Kehoe, 2010; Gutu and Kehoe, 2012; Hirose et al., 2013). RcaE has autokinase activity under RL and phosphorylates RcaF under these conditions, which in turn activates RcaC through phosphorylation. The phosphorylated form of RcaC activates transcription of PC biosynthetic genes; while simultaneously repressing transcription of PE biosynthetic genes under RL (Li et al., 2008; Bezy and Kehoe, 2010). In contrast, reduced kinase activity (Hirose et al., 2013) and/or phosphatase activity (Kehoe and Grossman, 1997) of RcaE under GL results in accumulation of RcaF and RcaC in unphosphorylated states, which results in transcriptional activation of PE biosynthetic genes due to de-repression, whereas PC biosynthetic genes are not expressed due to lack of accumulation of phosphorylated RcaC (Li et al., 2008; Bezy and Kehoe, 2010).

In addition to altering the pigment composition of PBSs, *F. diplosiphon* alters its cellular morphology and filament length during CCA to maximally utilize available resources (Montgomery, 2008). Rectangular cell shape and longer filaments are characteristics of morphological acclimation under GL, whereas spherical cell shape and shorter filaments are observed under RL (Bennett and Bogorad, 1973; Bogorad et al., 1983; Bordowitz and Montgomery, 2008). The molecular mechanism and ecological significance of light signaling-coupled alterations in pigmentation are well understood; however, the mechanism(s) and significance of morphological regulation that occurs during CCA are still emerging. One of the initial insights in this regard was the finding that photosensor RcaE controls the light-dependent regulation of *F. diplosiphon* morphology (Bordowitz and Montgomery, 2008). However, photoregulation of morphology was found to be largely independent of the photoregulation of pigmentation under GL and RL (Bordowitz and Montgomery, 2008; Bordowitz et al., 2010; Pattanaik et al., 2011). Recently, a correlation between RL-associated high levels of reactive oxygen species (ROS) and spherical morphology was established (Singh and Montgomery, 2012; Singh et al., 2013). GL-specific elongated cellular morphology has been proposed to provide greater cellular volume to support increased thylakoid membranes and light-harvesting complex capacity which would support efficient absorption of dim light and available GL photons in benthic environments (Montgomery, 2008). This would accommodate higher cellular amounts of PE, which is the sole GL-absorbing pigment (Campbell, 1996), and thus would allow maintenance of photosynthesis in benthic waters. Recent studies have supported this proposition, *F. diplosiphon* cells adopt a longer, rectangular shape in reduced light intensity and a spherical morphology in high light intensity, independent of whether the high intensity light is RL or GL (Pattanaik et al., 2012; Walters et al., 2013).

Recently, we conducted gene expression and gene function analyses in *F. diplosiphon* with an emphasis on the role of *bolA* and *mreB* morphogenes in the mechanistic bases of light-regulated morphological changes during CCA (Singh and Montgomery, 2014). Morphogene *bolA* was first identified in *Escherichia coli* by its ability to induce spherical morphology (Aldea et al., 1988). Stationary phase-dependent induction of spherical morphology was later shown to be associated with overexpression of *bolA* (Aldea et al., 1989). The expression of *bolA* is also upregulated in the presence of different stressors which are correlated with the induction of rod-to-spherical morphology changes of *E. coli* (Santos et al., 1999). The role of *bolA* in adaptation of bacterial systems to general stress has been explored in a recent review (Guinote et al., 2014).

BolA-dependent induction of spherical morphology in *E. coli* is known to be mediated, at least in part, by downregulation of transcription of the *mreB* gene through binding of BolA to the promoter region of *mreB* (Aldea et al., 1988, 1989; Santos et al., 1999; Freire et al., 2009). MreB is a bacterial actin-like ATPase, whose accumulation in bacterial cells is associated with rod-shape morphology and its absence with spherical cells (Cabeen and Jacobs-Wagner, 2007). The identification and functional characterization of BolA and MreB proteins in cyanobacteria and their role in maintaining cyanobacterial morphology have only recently begun to emerge (Hu et al., 2007; Gonzalez et al., 2010; Singh and Montgomery, 2014).

RcaE, which controls light-dependent regulation of cell shape (Bordowitz and Montgomery, 2008), was found to be required for normal WT expression of *bolA* (Singh and Montgomery, 2014). RL-dependent higher expression of *bolA* was correlated with lower expression of *mreB*; a converse relation was observed under GL (Singh and Montgomery, 2014). Lower expression of *mreB* under RL was proposed to be due to binding of accumulated BolA to the *mreB* promoter, which eventually results in spherical morphology due to decreased accumulation of MreB. In contrast, lower accumulation of BolA under GL permits higher expression of *mreB* which results in rod-shaped cell morphology due to higher accumulation of MreB (Singh and Montgomery, 2014).

To test whether RcaE-dependent regulation of cellular BolA protein abundance is a mechanism for photoregulation of cellular morphology, we generated antibodies against *F. diplosiphon* BolA and investigated whether known *bolA* expression patterns coincide with accumulation of BolA protein under GL and RL in *F. diplosiphon*. We also applied gene overexpression to probe whether elevated levels of BolA accumulation are correlated with induction of RL-characteristic spherical morphology independent of external light conditions and independent of the presence of functional RcaE to probe the molecular basis of morphological defects commonly observed in Δ*rcaE* null mutants.

## Materials and Methods

### Cyanobacterial Strains and Growth Conditions

The wild-type *F. diplosiphon* UTEX 481, hereafter UTEX 481; shortened-filament, wild-type pigmentation strain SF33 (Cobley et al., 1993), hereafter denoted SF33 WT; and RcaE-deficient mutant strain, i.e., Δ*rcaE* (Kehoe and Grossman, 1996), were used in this study. Strains were grown in BG-11 medium (Allen, 1968) containing 20 mM HEPES at pH 8.0 (hereafter BG-11/HEPES) in a GL growth chamber at an intensity of ∼15 μmol m^-2^ s^-1^ with continuous shaking at 175 rpm at 28°C. Exponentially growing cultures, which were diluted to an initial optical density of ∼0.2 at 750 nm (OD_750_), were then transferred to either GL or RL at an intensity of ∼15 μmol m^-2^ s^-1^ at 28°C with continuous shaking at 175 rpm. The sources of monochromatic GL and RL were those previously described (Bordowitz and Montgomery, 2008). The intensity of the light was measured with a LI-250A light meter (LI-COR, Lincoln, NE) equipped with a quantum sensor (model LI-190SA).

### Overexpression of WT *bolA* Gene in WT and *ΔrcaE* Background Strains

The overexpression of *bolA* in WT and Δ*rcaE* background strains under the control of the native promoter of the *apcA* gene, which encodes the α-subunit of allophycocyanin (Capuano et al., 1991), was achieved using the pPL2.7GW shuttle vector (Bordowitz and Montgomery, 2008). The promoter sequence of *apcA*, hereafter *apcAp*, and coding sequence of *bolA* were amplified from the *F. diplosiphon* genome using primer sets *apcAp*_FP/*apcAp*_RP and *bolA*_FP/*bolA*_RP, respectively. All primer sequences used in this study are listed in **Supplemental Table S1**. The resulting PCR products for the 284 bp *apcAp* sequence and 272 bp *bolA* coding sequence were gel purified, and 70 ng of each product was mixed together for overlap PCR. A 1.5 μl aliquot of this mixture was used as a template in a new 50 μl overlap PCR reaction with primers *apcAp*_FP and *bolA*_RP to add the *apcAp* promoter to the *bolA* full-length gene. The 526 bp *apcAp-bolA* fusion PCR product was verified by sequencing. The verified fusion product was cloned into PCR^TM^8/GW/TOPO^®^ vector (Invitrogen) according to the manufacturer’s protocol to produce entry vector PCR^TM^8/GW/TOPO-*apcA*p-*bolA*, and subsequently used to transform TOP10 chemically competent cells. Following selection of transformants in the presence of spectinomycin, PCR^TM^8/GW/TOPO-*apcA*p-*bolA* was isolated and thereafter recombined with pPL2.7GW (Bordowitz and Montgomery, 2008). All plasmids used or constructs made in this study are listed in **Supplemental Table S2**. The recombination was achieved by using the LR clonase II enzyme according to the manufacturer’s instruction (Invitrogen) to produce an expression vector pPL2.7GW-*apcA*p-*bolA*, which was used to transform DH5α cells. Transformants were selected in the presence of kanamycin. *E. coli* strains used in this study were grown overnight in liquid LB (Luria-Bertani) medium or on LB agar plates, i.e., LB solidified with the addition of 1.5% (w/v) agar, at 37°C. When indicated, antibiotics were added at the following concentration: spectinomycin at 100 μg/ml (w/v) or kanamycin at 50 μg/ml (w/v). pPL2.7GW-*apcA*p-*bolA* was transformed into WT and Δ*rcaE* strains via electroporation as described previously (Kehoe and Grossman, 1998) to obtain WT overexpression (OE) and Δ*rcaE* OE strains overexpressing *bolA*. Empty shuttle vector pPL2.7 (Chiang et al., 1992) was used during transformation as a positive control, whereas autoclaved double distilled water was used as a negative control. WT OE and Δ*rcaE* OE strains were grown in BG-11/HEPES medium supplemented with 3.5 and 6 μg/ml concentration of kanamycin, respectively.

### Cell Density Measurements and Spectral Scans

Growth of different strains of *F. diplosiphon* was monitored based on cell density estimated based on optical density at 750 nm (OD_750_) using a SpectraMax M2 microplate reader (Molecular Devices, Sunnyvale, CA, USA). Whole-cell spectral scans were obtained between 400 and 800 nm after adjusting the OD_800_ of cell cultures to ∼0.1 using a SpectraMax M2 microplate reader.

### Chlorophyll *a* (chl *a*) and Phycobiliprotein Quantification Assays

Chlorophyll *a* (chl *a*) was extracted from 1 ml of culture after 7 days of growth and quantified using a previously reported method (De Marsac and Houmard, 1988) with modifications as previously detailed (Singh and Montgomery, 2011). PBPs were extracted from 1 ml of cell culture after 7 days of growth as described (Kahn et al., 1997) with modifications previously detailed (Singh and Montgomery, 2011), except that the extraction was conducted for 1 h. PBP levels were calculated using equations from De Marsac and Houmard (1988).

### Reactive Oxygen Species (ROS) Quantification Assay

Cellular levels of ROS were determined using the fluorescent dye 2′,7′-dichlorodihydrofluorescein diacetate (DCFH-DA; EMD chemicals) as previously described (Singh and Montgomery, 2012). Immediately after sample collection, the cells were incubated with 10 μM DCFH-DA (final concentration) in the dark for 1 h at room temperature with continuous shaking, and thereafter, DCF fluorescence was measured at 520 nm after excitation at 485 nm using a SpectraMax M2 microplate reader. Growth medium mixed with DCFH-DA dye acted as a negative control. Fluorescence from cellular components at 520 nm after excitation at 485 nm was measured using samples containing only cells and lacking DCFH-DA dye.

### Confocal Microscopy-based Morphological Analyses

*Fremyella diplosiphon* cultures were inoculated at an OD_750_ of ∼0.2 and grown under GL and RL. After 3 days of growth, slides of live, immobilized *F. diplosiphon* cells were prepared as previously described (Bordowitz and Montgomery, 2008). Images of cells were acquired with an inverted Axiovert 200 Zeiss LSM 510 Meta confocal laser scanning microscope (Carl Zeiss MicroImaging, Thornwood, NY, USA) using differential interference contrast (DIC) optics and fluorescence excitation and emission filters as described earlier (Bordowitz and Montgomery, 2008, 2010). The length and width of cells (*n* = 50) and filament length (*n* = 30–50) were measured using LSM FCS Zeiss 510 Meta AIM imaging software.

### Production of anti-BolA Antibodies and Affinity Purification

Purified *F. diplosiphon* (His)_6_-BolA protein was obtained as described previously (Singh and Montgomery, 2014). Purified protein (2.5 mg), which had been heated at 90°C for 5 min in 1X SDS sample buffer containing 5% (v/v) β-mercaptoethanol (i.e., reducing conditions), was separated on a 1-mm thick 15% (w/v) SDS-polyacrylamide gel and stained with Coomassie stain. Following staining, the BolA protein band was sliced from the gel and destained overnight. Thereafter, the (His)_6_-BolA-containing gel slice was frozen in liquid nitrogen and kept at -80°C until submitted to Pacific Immunology (Ramona, CA, USA) for the production of anti-(His)_6_-BolA antibodies in New Zealand white rabbits. The crude antiserum obtained was tested against purified (His)_6_-BolA using western blot analysis, and further affinity purification was performed against purified (His)_6_-BolA protein to obtain highly specific anti-BolA antibodies. Briefly, ∼300 μg of purified (His)_6_-BolA protein was run on a 15% (w/v) SDS-polyacrylamide gel and transferred to PVDF membrane by electro-blotting (see details for transfer during western blot analyses below). The transfer was evaluated by Ponceau S staining, and the portion of the membrane containing the BolA protein band was extracted. This membrane slice containing (His)_6_-BolA was incubated for 1 h at room temperature in TBS (20 mM Tris-HCl, pH 7.5, 150 mM NaCl) containing 0.1% (v/v) Tween 20 and 5% (w/v) BSA. The blot was then incubated overnight at 4°C on a rocking platform with crude antiserum, the latter of which was previously saturated with BSA (12 mg/ml; w/v) at 37°C for 4 h. Following this incubation, the blot was washed 3 × 15 min with TBS/0.1% Tween 20 followed by one additional washing with TBS. After washing, the blot was cut into small pieces and adsorbed antibodies eluted with 700 μl of 0.2 M HCl-glycine, pH 2.2, at room temperature for 15 min. Thereafter, the pH of the eluate was adjusted to pH 7 by adding 300 μl of 1M K_2_HPO_4_. The neutralized eluate was dialyzed overnight at 4°C against TBS using 10 KDa cut-off Snakeskin dialysis tubing (Thermo Scientific). After dialysis, 5% BSA was added to the eluted anti-BolA antibodies and aliquots were kept at -20°C for further use.

### Total Protein Extraction and Western Blot Analyses

Liquid cultures (50 ml) of *F. diplosiphon* were started at an OD_750_ of 0.2 and grown under GL or RL growth conditions for 7 days to an OD_750_ of ∼0.7–0.9. Cells were flash chilled to ∼4°C in a flask by submersion in liquid nitrogen, then pelleted by centrifugation at 5000 *g* for 10 min at 4°C. Cell pellets were resuspended in 2 ml of CellLytic^TM^ B cell Lysis reagent supplemented with 1 × SIGMAFAST^TM^ protease inhibitor cocktail (Sigma–Aldrich). Cells were lysed by incubating the suspension at room temperature for 20 min with regular vortexing. After cell lysis, the extract was centrifuged at 16000 *g* for 10 min at 4°C to pellet the insoluble material, and supernatant was transferred to a new Eppendorf tube. Total protein concentrations of different samples were determined using a Pierce^TM^ BCA protein assay kit (Thermo Scientific). Proteins, which had been heated at 90°C for 5 min in 1X SDS sample buffer containing 5% (v/v) β-mercaptoethanol, were separated on a 1-mm thick 15% (w/v) reductive SDS-polyacrylamide gel and transferred onto Immobilon^®^-P polyvinylidene difluoride membrane (pore size 0.45 μm; Millipore, Billercia, MA, USA) via electro-blotting using a Trans Blot Turbo transfer system (Bio-Rad, Hercules, CA, USA) at 20 V, 1 A for 10 min at 24°C according to manufacturer’s instructions. To detect BolA, the membrane was incubated in a blocking solution (TBS/0.5% Tween 20/3% BSA) for 1 h at room temperature. Following blocking, immunoblot was incubated overnight at 4°C with affinity purified anti-BolA polyclonal antibodies in blocking solution (1:1000 dilution). After washing the blot 4 × 10 min with washing buffer (TBS/0.1% Tween 20), it was incubated for 3 h at room temperature with anti-rabbit secondary antibodies conjugated to horseradish peroxidase (Pierce Biotechnology, Inc., Rockford, IL, USA) that was diluted (1:10,000) in blocking solution. Following 4 × 10 min washes with washing buffer, signal was detected using WesternBright^TM^ ECL western blotting detection kit (Advansta, Menlo Park, CA, USA) on a Molecular Imager VersaDoc MP 4000 imaging system (Bio-Rad, Hercules, CA, USA).

### Biofilm Formation Assay

Biofilm formation by *F. diplosiphon* WT and WT OE strains under GL and RL was assessed according to the method described previously with minor modifications (O’Toole and Kolter, 1998). Briefly, 1-ml aliquots of actively growing cultures of WT and WT OE strains, which were adjusted to an OD_750_ of 0.5 in fresh medium, were transferred in triplicate into wells of a Greiner Bio-One CELLSTAR^®^ 24-well polystyrene cell culture plate (BioExpress, Kaysville, UT, USA). BG11 medium without cells was added in triplicate as a control. Plates were transferred to GL and RL growth conditions with gentle shaking. On the fourth day after transfer, medium was removed from each well, wells were washed three times with autoclaved double-distilled water to remove unattached cells, and plates were air-dried for 30 min. Biofilm was stained for 30 min with 0.25% (w/v) crystal violet in water, after which the stain was removed and wells were washed three times with water. The stain in attached biofilms was extracted in 500 μl of 100% ethanol for 15 min, and OD_595_ measured to quantify the biofilm formation.

### Statistics

All experiments were conducted with three replicates and results are presented as the mean value (±SD). Data were analyzed by a one-way ANOVA test using OpenStat statistical software [version 10.01.08; (Miller, 2013)]. Once a significant difference was detected, *post hoc* multiple comparisons were made using the Tukey test. The significance level was set at 0.05 for all statistical analyses.

## Results

### BolA Levels are Regulated Transcriptionally in an RcaE-dependent Manner

**FIGURE 1:**
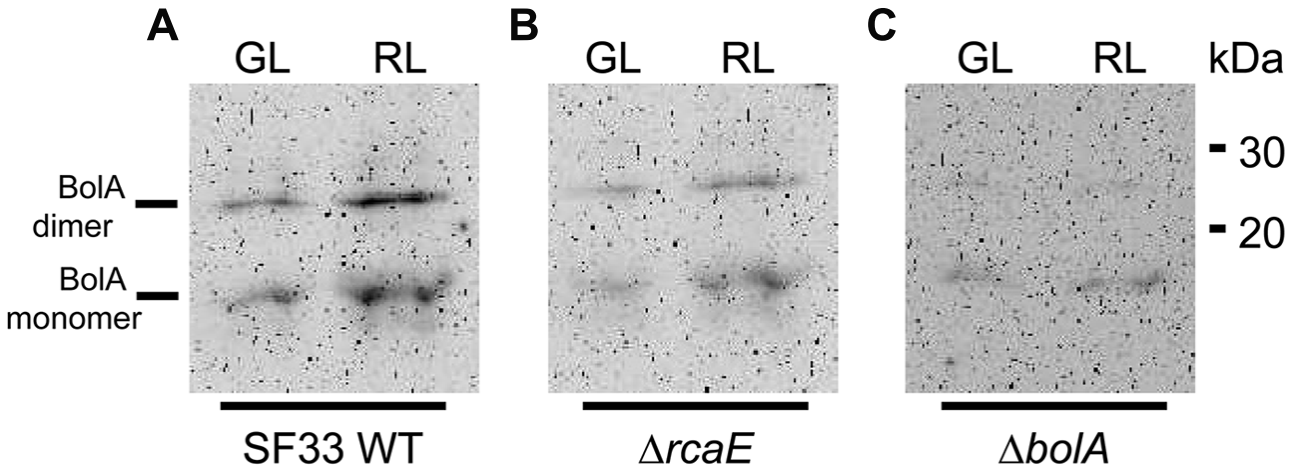
**Immunoblot analyses of BolA accumulation in *Fremyella diplosiphon* strains.** BolA accumulation in **(A)** WT, **(B)** Δ*rcaE*, and **(C)** Δ*bolA* strains of *F. diplosiphon* under green light (GL) or red light (RL). 150 μg of total protein extract from different strains of *F. diplosiphon* were separated on 15% SDS-PAGE. After blotting, BolA was detected using affinity-purified anti-BolA antibodies. Molecular mass in kilodalton (kDa) is indicated to the right. BolA monomer (∼11 kDa) and putative dimer (∼22 kDa) are marked to the left.

Prior analyses indicated that photoregulation of *bolA* mRNA levels is inversely correlated with accumulation of bacterial-actin encoding *mreB* mRNA levels, which is associated with light-dependent regulation of cellular morphology in *F. diplosiphon* (Singh and Montgomery, 2014). We generated antibodies against *F. diplosiphon* BolA and examined accumulation of BolA under GL and RL in different strains of *F. diplosiphon* in order to investigate whether *bolA* mRNA levels reflect BolA protein accumulation. In accordance with previous observations for *bolA* mRNA accumulation under GL and RL in WT (Singh and Montgomery, 2014), BolA protein was found to accumulate to higher levels in cells under RL than in GL (**Figure 1A**). FdBolA is present as both a monomer and dimer, as previously observed for BolA (Couturier et al., 2014). The monomeric form has been reported as a glutathionylated form, the dimeric form contains a disulfide bridge, and the monomer:dimer ratio can change during incubation or storage (Couturier et al., 2014). We also examined levels of BolA under GL and RL in Δ*bolA* and Δ*rcaE* strains, which previously were found to have lower accumulation of *bolA* mRNA relative to WT (Singh and Montgomery, 2014). Both Δ*bolA* and Δ*rcaE* strains were found to accumulate low levels of BolA protein under GL and RL in comparison to WT (**Figure 1**). BolA accumulation thus appears to be largely regulated transcriptionally (**Figure 1**; Singh and Montgomery, 2014). Furthermore, functional RcaE promotes light-dependent BolA accumulation under GL and RL, indicating that RcaE-dependent higher expression of *bolA* under RL results in higher accumulation of BolA protein.

### BolA Overaccumulation Induces Spherical Morphology and Long Filaments Independent of the External Light Conditions

**FIGURE 2:**
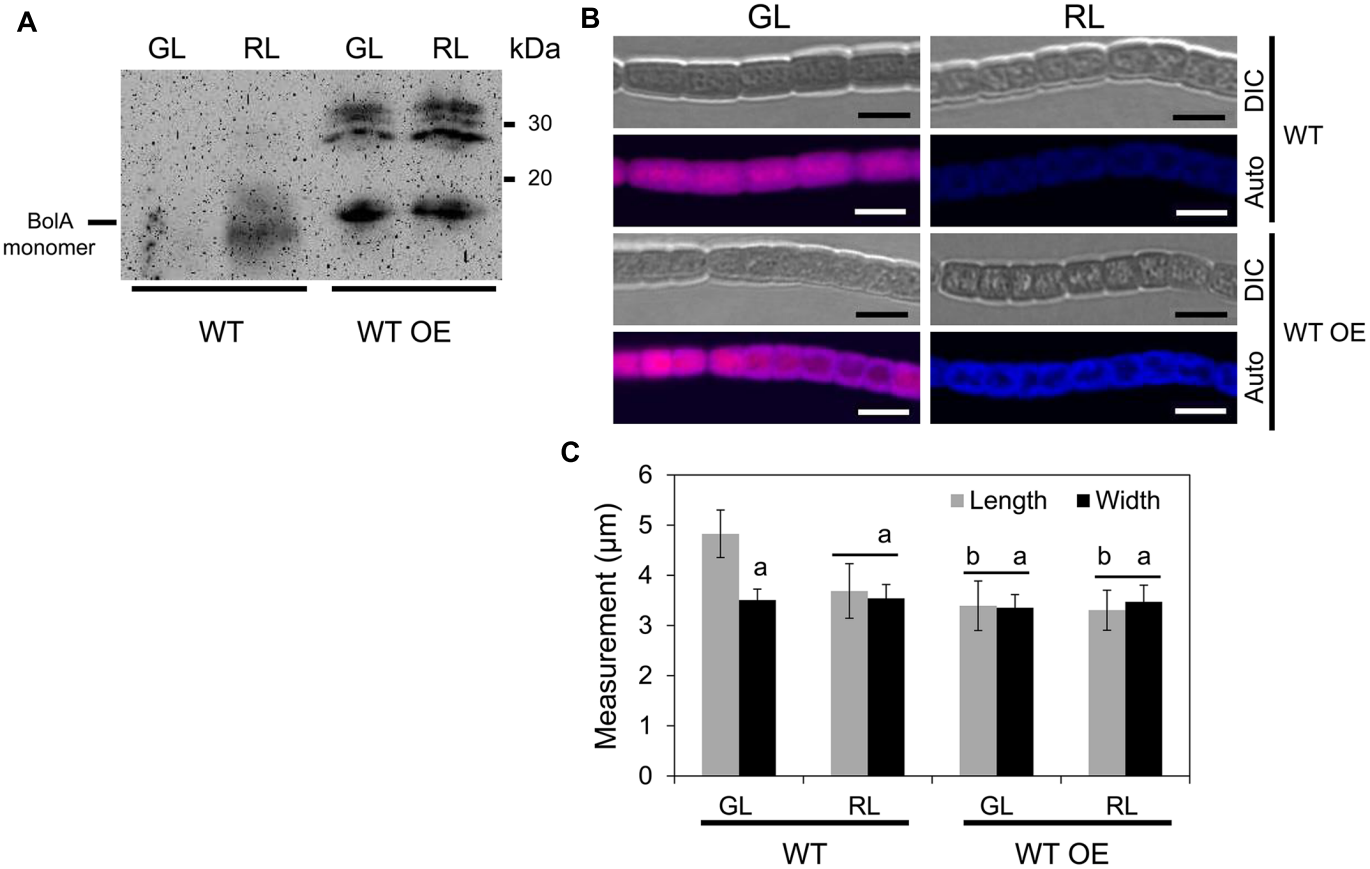
**Immunoblot analysis of BolA accumulation and confocal laser scanning microscopy analyses of the cellular morphology of *F. diplosiphon* wild-type (WT) and WT strain overexpressing *bolA* (WT OE) grown under green light (GL) or red light (RL).** **(A)** 150 μg of total protein extract from different strains of *F. diplosiphon* grown under GL or RL were separated on 15% SDS-PAGE. After blotting, BolA was detected using affinity-purified anti-BolA antibodies. Molecular mass in kilodalton (kDa) is indicated to the right. **(B)** Representative optical slices from a Z-series of differential interference contrast (DIC) images and corresponding maximum intensity projection PBP autofluorescence (auto) images of WT and WT OE strains grown under GL or RL for 72 h. Images were acquired using a 40×oil immersion objective with 2× zoom setting. Bars, 5 μm. **(C)** Cell length and width measurements of *F. diplosiphon* WT and WT OE strains grown under GL or RL for 72 h. Identical letters over bars represent a homogenous mean group (*P* > 0.05), whereas line over bars indicate no significant difference between the length and width of cells (*P* > 0.05) for a particular condition. No symbol over the bar indicates a significant difference (*P* < 0.05) from others.

**FIGURE 3:**
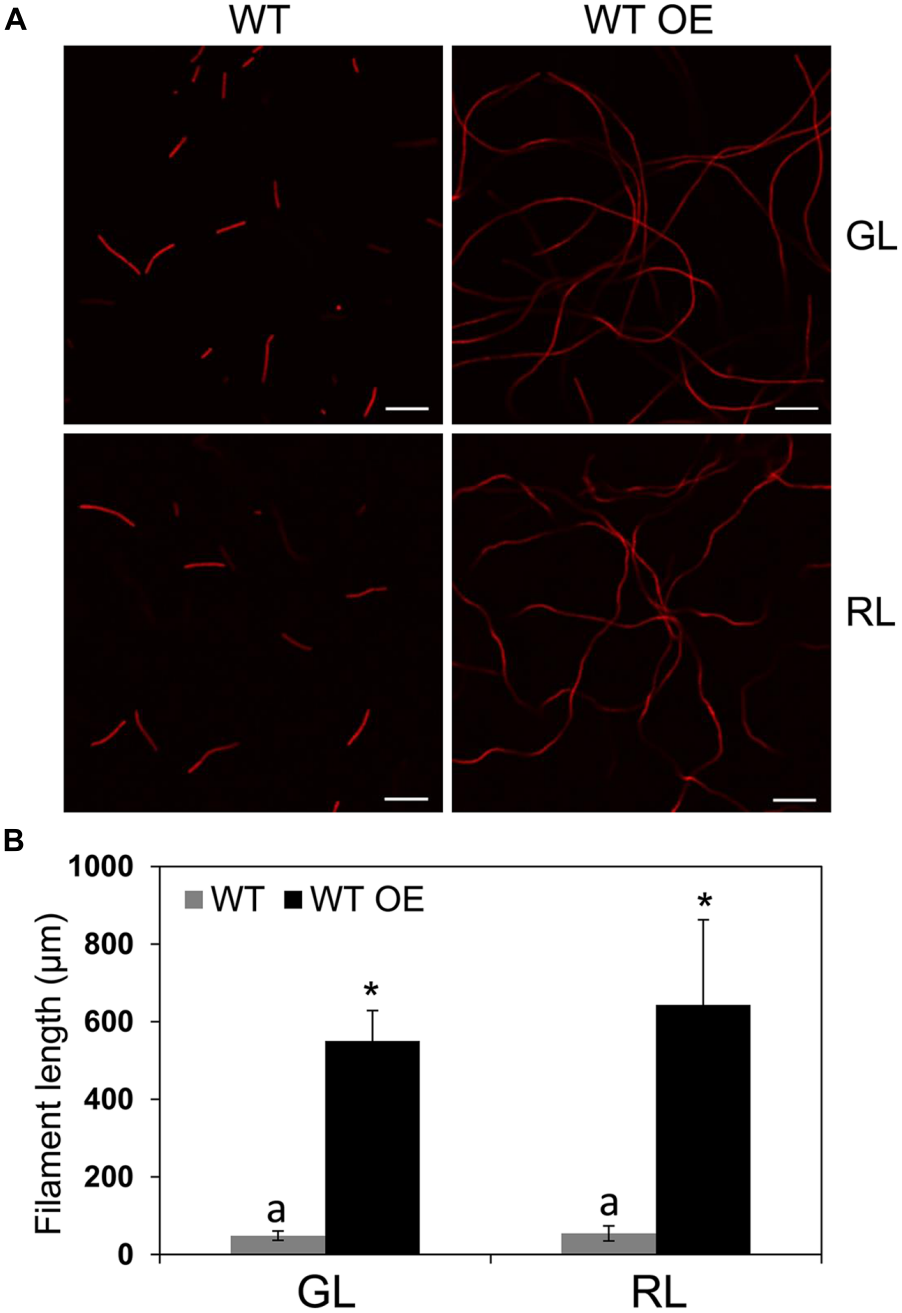
**Confocal laser scanning microscopy analyses of filament morphology of *F. diplosiphon* wild-type (WT) and WT strain overexpressing *bolA* (WT OE) grown under GL or RL.** **(A)** Representative optical slices from a Z-series of chlorophyll autofluorescence images of WT and WT OE strains grown under GL and RL for 72 h. Images were acquired using a 10× objective with 2× zoom setting. Bars, 50 μm. **(B)** Filament length measurements of WT and WT OE strains grown under GL or RL for 72 h. Identical letters over bars represent a homogeneous mean group (*P* > 0.05), whereas asterisks indicate a significant difference (*P* < 0.05) from WT.

To probe the specific role of BolA in photoregulation of morphology during CCA by altering *bolA* copy number, we overexpressed *bolA* in *F. diplosiphon*. We first attempted to overexpress *bolA* under the control of its endogenous promoter (i.e., np*bolA*) in WT; however, an np*bolA*-overexpressing (OE) strain was found to accumulate lower levels of BolA compared to the WT strain (**Supplemental Figure S1**). Therefore, we overexpressed *bolA* under the control of the promoter sequence of the *apcA* gene, which is known to be expressed equally under GL and RL and at relatively high levels in cells (Oelmüller et al., 1988; Capuano et al., 1991). In comparison to the WT strain that was transformed with an empty vector control (hereafter designated WT) and for which we observed more monomer than dimer, the WT strain overexpressing *bolA* gene under the control of the *apcA* promoter (hereafter designated WT OE) accumulated higher levels of BolA than WT under GL and RL (**Figure 2A**). The overexpression of *bolA* resulted in induction of a RL-associated spherical morphology of cells in the WT OE strain under both GL and RL, whereas cells of the WT strain possessed WT-characteristic rectangular shape under GL and spherical morphology under RL which was similar to that previously reported for *F. diplosiphon* (**Figure 2B**; Bennett and Bogorad, 1973; Bordowitz and Montgomery, 2008). Similar to previous reports, decreased cell length and no significant difference between length and width of the cells were observed under RL, whereas the length of cells was greater than width under GL, which corresponded to a rectangular morphology of WT cells (**Figure 2C**; Bordowitz and Montgomery, 2008; Singh and Montgomery, 2012). Higher accumulation of BolA in the WT OE strain was associated with a decrease in the length of cells, and no significant difference between length and width of cells was observed under GL or RL (**Figure 2C**). In addition to an effect on cell shape, higher BolA accumulation in the WT OE strain unexpectedly resulted in an increase in filament length under both light conditions, whereas the filament length of WT under GL and RL was comparable to previous observations (**Figure 3**; Bordowitz and Montgomery, 2008).

### BolA Overaccumulation Reduces ROS Accumulation

**FIGURE 4:**
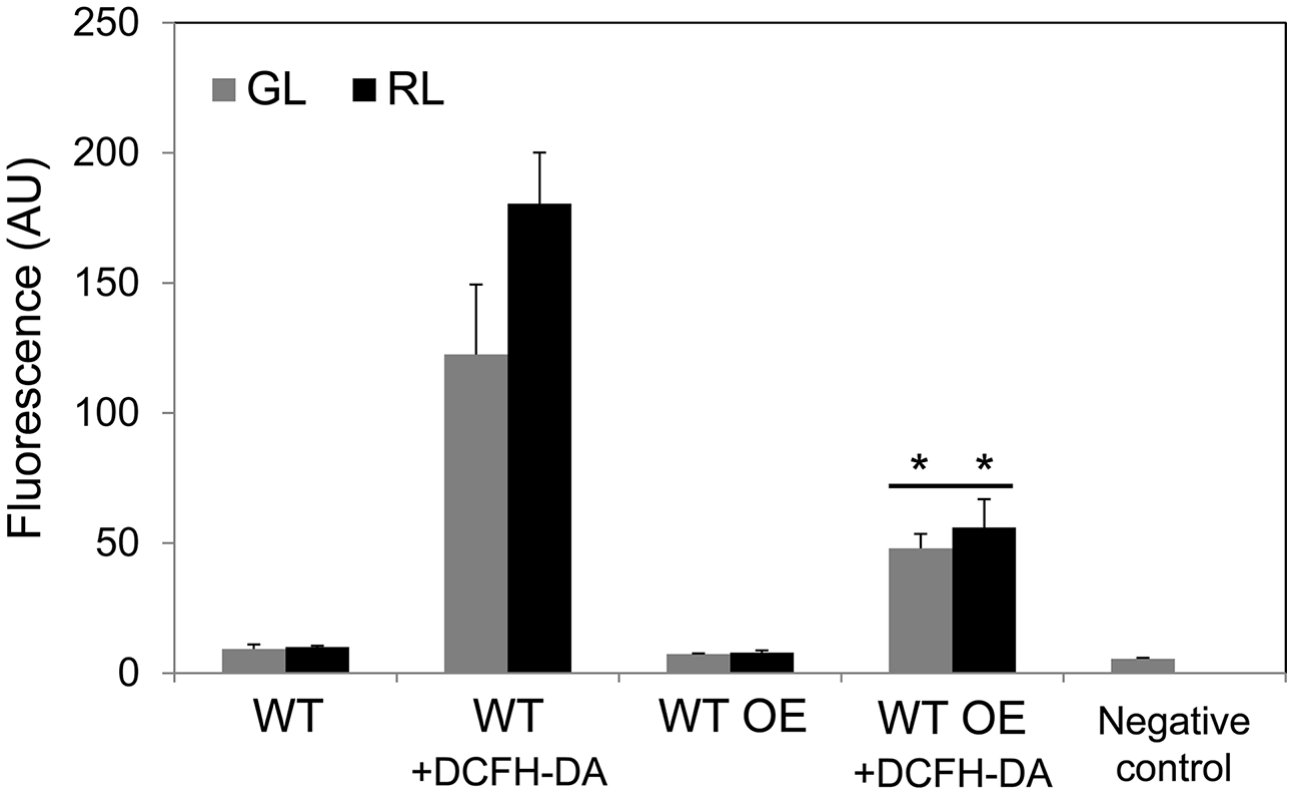
**Reactive oxygen species (ROS) accumulation in *F. diplosiphon* wild-type (WT) and WT strain overexpressing *bolA* (WT OE) grown under GL or RL.** Cellular component-associated fluorescence at 520 nm or ROS-dependent dichlorodihydrofluorescein (DCF) fluorescence at 520 nm in arbitrary units (AU) after 72 h of growth of cells with DCFH-DA dye added (+DCFH-DA) under GL or RL. The negative control represents fluorescence originating from BG-11 medium + ROS-sensitive dye DCFH-DA without cells added. Asterisks indicate significant difference (*P* < 0.05) from WT counterpart grown under GL or RL, whereas line over bars indicate no significant difference (*P* > 0.05) between GL- and RL- grown samples of a particular strain.

Removal of all but a few copies of *bolA* in a partially segregated Δ*bolA* strain (a complete null was lethal; Singh and Montgomery, 2014) resulted in significantly higher levels of ROS under GL and RL, which suggested an involvement of BolA in controlling intracellular ROS levels (Singh and Montgomery, 2014). Notably, a higher accumulation of BolA in the WT OE strain was associated with significantly decreased levels of ROS under GL and RL in comparison to WT (**Figure 4**). In WT, ROS levels were higher under RL compared to GL (**Figure 4**), as reported earlier (Singh and Montgomery, 2012; Singh et al., 2013).

### BolA Overexpression does not Induce Biofilm Formation in Filamentous *F. diplosiphon*

**FIGURE 5:**
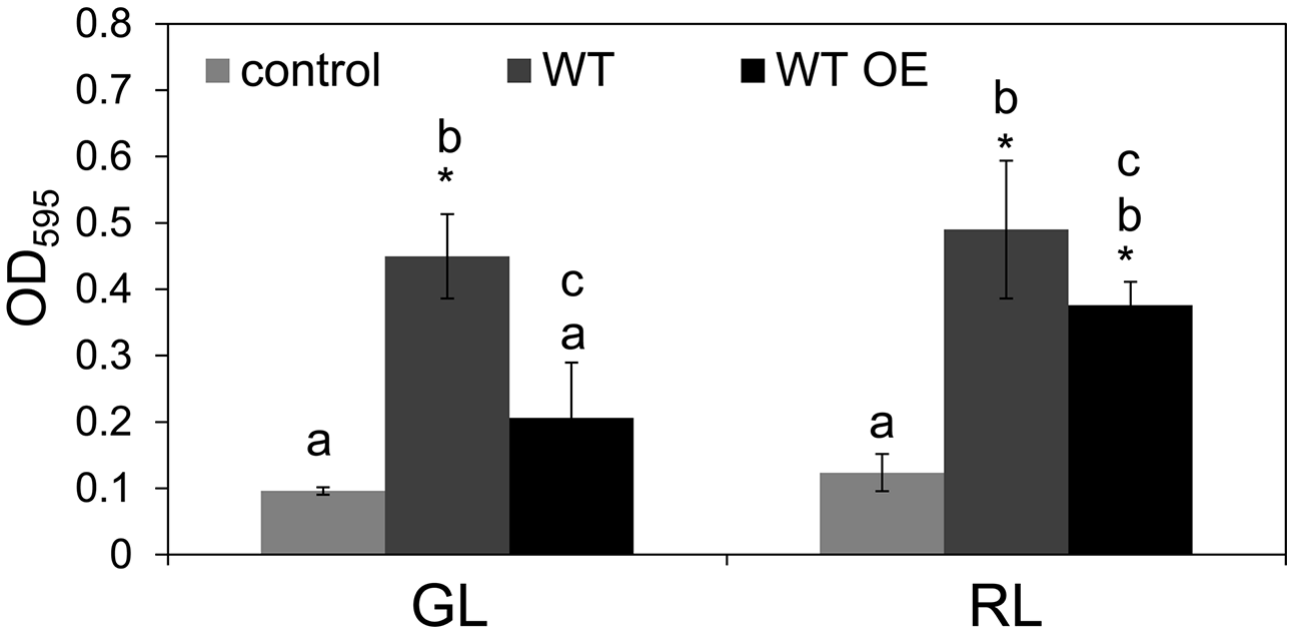
**Biofilm development in *F. diplosiphon* wild-type (WT) and WT strain overexpressing *bolA* (WT OE) under GL and RL growth conditions.** Biofilm formation was measured as optical density at 595 nm (OD_595_) after staining with crystal violet. Bars represent average (±SD) from three independent biological replicates. Asterisks indicate significant difference (*P* < 0.05) from control samples, whereas identical letters over bars represent homogenous mean groups (*P* > 0.05).

Overexpression of native *bolA* or *bolA* from distantly related organisms, including *F. diplosiphon*, was found to induce an increased level of biofilm formation and spherical morphology in *E. coli* (Vieira et al., 2004; Freire et al., 2009; Khona et al., 2013; Singh and Montgomery, 2014). These observations suggested that BolA function is conserved and prompted us to examine whether BolA accumulation in *F. diplosiphon* is associated with an increased level of biofilm formation. However, we found that a higher level of BolA accumulation in the WT OE strain was correlated with a decreased level of biofilm formation in comparison to the WT strain under both GL and RL (**Figure 5**), distinct from its overexpression in a unicellular *E. coli* strain (Singh and Montgomery, 2014). Noticeably, the longer filaments of WT OE strain predominantly collected at the surface of growth medium in comparison to the WT strain where a comparatively higher proportion of filaments were attached to the bottom of the plate. This difference was associated with an apparent significantly higher level of biofilm formation in WT strain than in WT OE (**Figure 5**).

### BolA Overaccumulation in WT has Minor Impacts on Growth or Pigmentation during CCA

We also conducted whole-cell spectral scans of WT and WT OE strains to test whether higher accumulation of BolA in the WT OE strain has any effect on growth or the CCA response of this strain. The WT OE strain accumulated GL- and RL-dependent PBPs PE and PC, respectively, similar to the WT strain under identical growth conditions (**Supplemental Figure S2A**). These results suggested that there was no alteration in the CCA response of the WT OE strain due to higher accumulation of BolA. This observation is in accordance with there being no significant impact on CCA related to a decreased gene dosage of *bolA* in the Δ*bolA* strain (Singh and Montgomery, 2014), which accumulates low levels of BolA under GL and RL (**Figure 1**). In comparison to WT, a higher accumulation of BolA in the WT OE strain did not impose any major quantitative effect on pigment accumulation under GL and RL. However, the WT OE strain accumulated a higher concentration of PE under GL compared to the WT (**Supplemental Figure S3**). No major effect of increased levels of BolA accumulation was seen on the growth of WT OE strain. However, the WT OE strain registered moderately increased growth under GL in comparison to WT after few days of growth estimation (**Supplemental Figure S2B**).

### BolA Overexpression in *ΔrcaE* Reverts Phenotypic Defects

**FIGURE 6:**
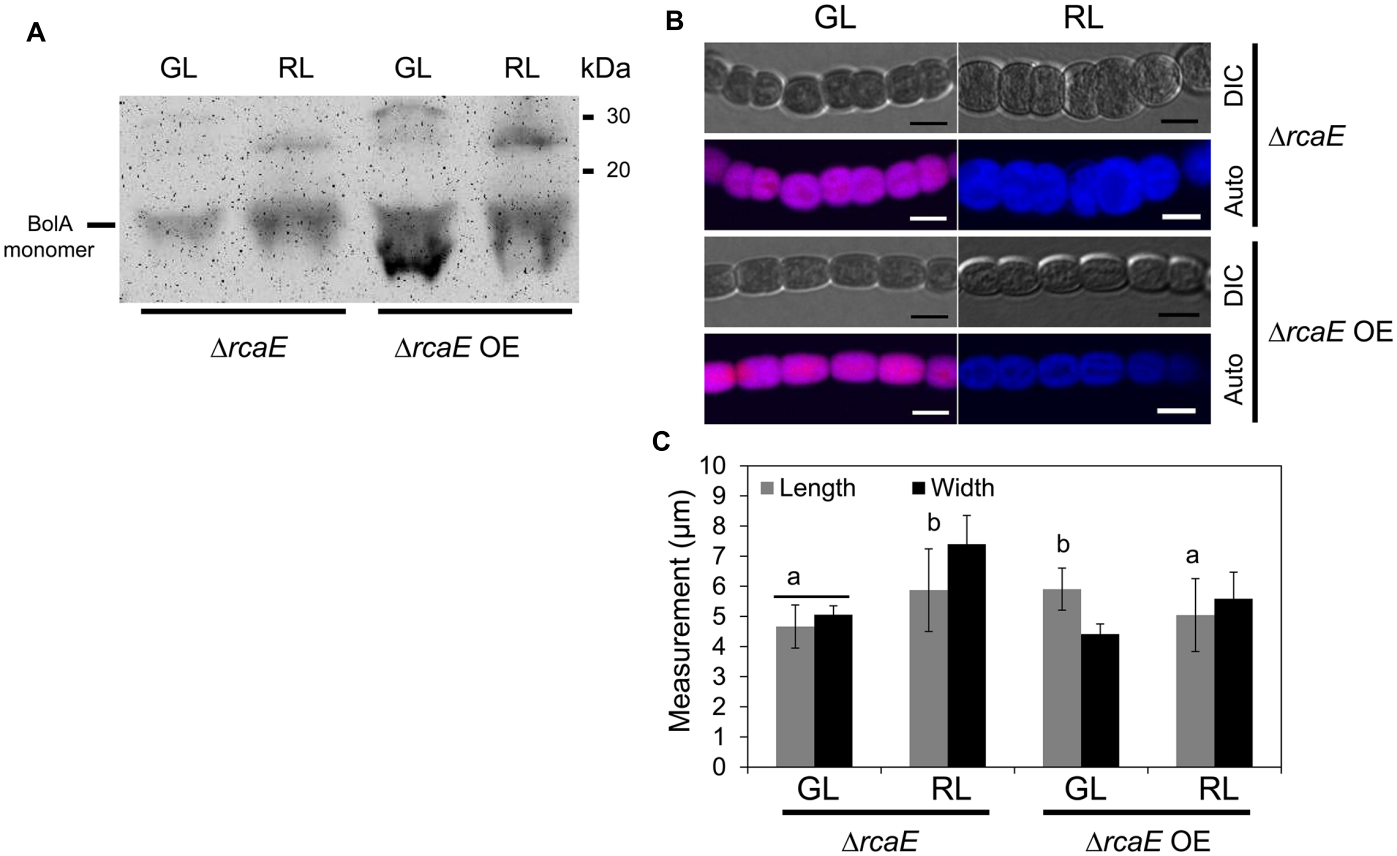
**Immunoblot analysis of BolA accumulation and confocal laser scanning microscopy analyses of the cellular morphology of *F. diplosiphon* Δ*rcaE* and Δ*rcaE* strain overexpressing *bolA* (Δ*rcaE* OE) grown under GL or RL.** **(A)** 300 μg of total protein extract from different strains of *F. diplosiphon* grown under GL or RL was separated on 15% SDS-PAGE. After blotting, BolA was detected using affinity-purified anti-BolA antibodies. Molecular mass in kilodalton (kDa) is indicated to the right. **(B)** Representative optical slices from a Z-series of DIC images and corresponding maximum intensity projection PBP autofluorescence (auto) images of Δ*rcaE* and Δ*rcaE* OE strains grown under GL or RL for 72 h. Images were acquired using a 40× oil immersion objective with 2× zoom setting. Bars, 5 μm. **(C)** Cell length and width measurement of *F. diplosiphon*Δ*rcaE* and Δ*rcaE* OE strains grown under GL or RL for 72 h. Identical letters over bars represent a homogenous mean group (*P* > 0.05), whereas line over bars indicate no significant difference between the length and width of cells (*P* > 0.05) for a particular condition. No symbol over the bar indicates a significant difference (*P* < 0.05) from others.

**FIGURE 7:**
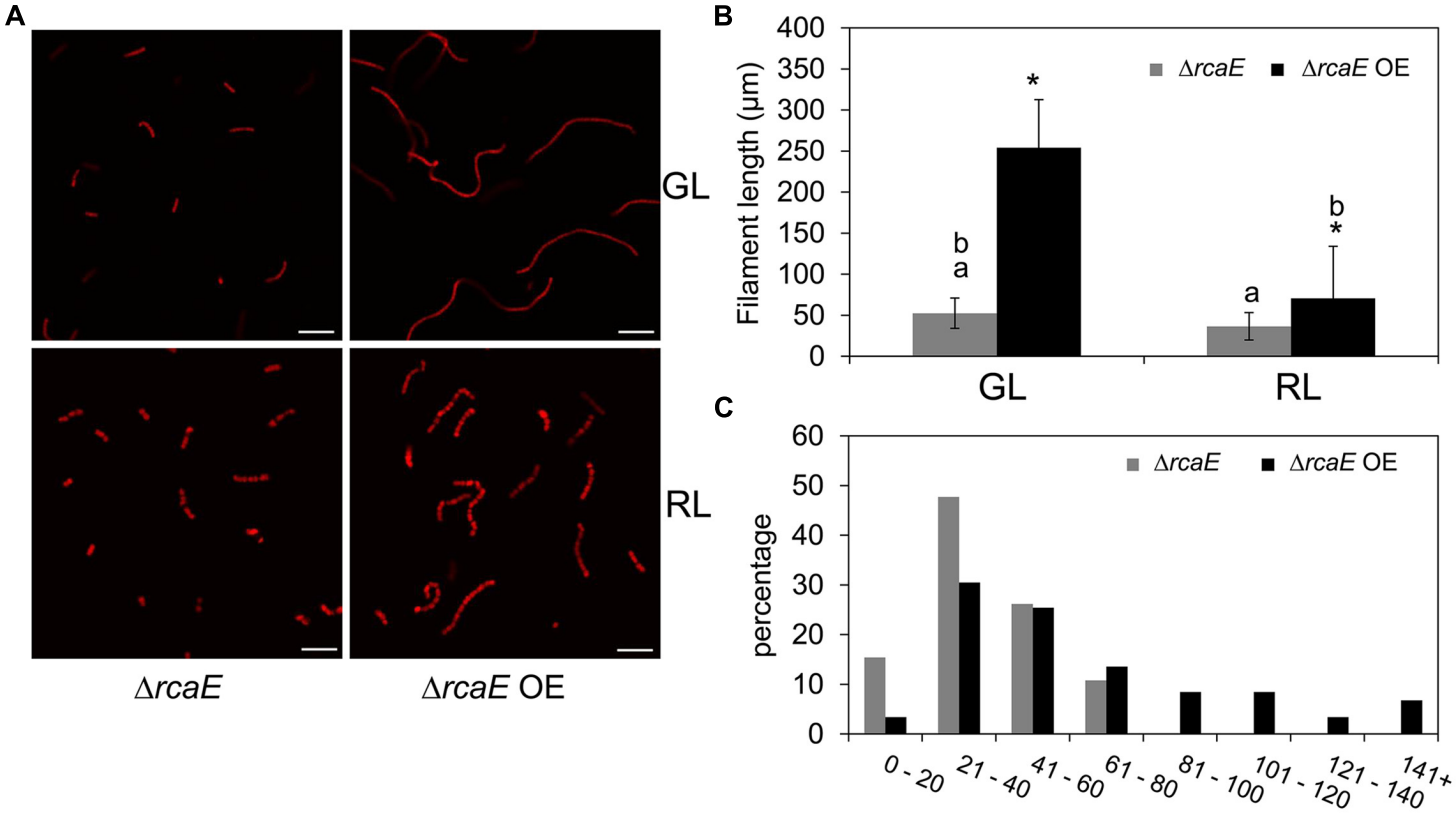
**Confocal laser scanning microscopy analyses of filament morphology of *F. diplosiphon* Δ*rcaE* and Δ*rcaE* strain overexpressing *bolA* (Δ*rcaE* OE) grown under GL or RL.** **(A)** Representative optical slices from a Z-series of chlorophyll autofluorescence images of Δ*rcaE* and Δ*rcaE* OE strains grown under GL or RL for 72 h. Images were acquired using a 10× objective with 2× zoom setting. Bars, 50 μm. **(B)** Filament length measurements of Δ*rcaE* and Δ*rcaE* OE strains grown under GL or RL for 72 h. Identical letters over bars represent a homogeneous mean group (*P* > 0.05), whereas asterisk indicates a significant difference (*P* < 0.05) from Δ*rcaE*. **(C)** Percentage distribution of filaments among indicated measured length (μm) in RL-grown cultures of Δ*rcaE* and Δ*rcaE* OE strains.

Observed low levels of *bolA* transcript and BolA accumulation under GL and RL in the Δ*rcaE* strain suggested that part of the morphological defects and high ROS levels commonly observed in Δ*rcaE* relative to WT could be associated with low accumulation of BolA in the absence of RcaE (Bordowitz and Montgomery, 2008; Singh and Montgomery, 2012, 2014). Our experimental attempt to overexpress the *F. diplosiphon bolA* gene in WT background strain under the control of its native promoter sequence resulted in a WT-np*bolA* strain with decreased accumulation of BolA in comparison to the WT (**Supplemental Figure S1**). We, thus, assessed whether this WT-np*bolA* strain exhibited an altered morphology associated with reduced BolA levels similar to Δ*rcaE*. Indeed, reduced BolA accumulation in this strain was associated with altered cellular morphology (**Supplemental Figure S4**), which was comparable to the known cellular morphology of the Δ*rcaE* strain that also exhibits reduced BolA levels (Bordowitz and Montgomery, 2008). To further probe the association of BolA accumulation in a Δ*rcaE* background with observed cellular morphology, we overexpressed *bolA* in the Δ*rcaE* null mutant (hereafter designated Δ*rcaE* OE). In comparison to Δ*rcaE*, the Δ*rcaE* OE strain accumulated higher levels of BolA under GL and RL (**Figure 6A**). Cells of the Δ*rcaE* null mutant are characterized by comparatively larger size and round shape morphology than WT under both GL and RL, which is apparently caused by an increase in width of Δ*rcaE* cells (Bordowitz and Montgomery, 2008; Singh and Montgomery, 2012). Notably, a higher accumulation of BolA under GL and RL in Δ*rcaE* OE strain was found to be associated with altered cellular morphology relative to the parent strain (**Figure 6B**). Increased levels of BolA in this Δ*rcaE* OE strain under GL was associated with a significant increase in length and a decrease in width of cells, which ultimately resulted in cells more rectangular in morphology than parental Δ*rcaE* cells (**Figures 6B,C**). The more rectangular morphology of Δ*rcaE* OE cells was much more similar, though not identical, to the known GL-specific cellular morphology of WT (compare **Figures 2B** and **6B**; Bordowitz and Montgomery, 2008; Singh and Montgomery, 2012). Higher accumulation of BolA in the Δ*rcaE* OE strain under RL was associated with significant decreases in length and width of cells in comparison to the RL-grown Δ*rcaE* strain (**Figure 6C**). No major difference was observed between length and width of cells under RL which resulted in cells that were still spherical though smaller in Δ*rcaE* OE than in Δ*rcaE* (**Figures 6B,C**). The higher accumulation of BolA in Δ*rcaE* OE strain under GL and RL was also associated with a significant increase in the length of filaments, which was similar to the observation made for the WT OE strain (**Figure 7**). However, a homogenous population of longer Δ*rcaE* OE filaments was observed under GL in comparison to the RL-grown cultures where a comparatively heterogeneous population of short and long filaments was observed (**Figure 7A**). A percentage distribution analysis of filaments of RL-grown Δ*rcaE* and Δ*rcaE* OE strains demonstrated that filaments longer than 80 μm were only observed in Δ*rcaE* OE strain (**Figure 7C**).

**FIGURE 8:**
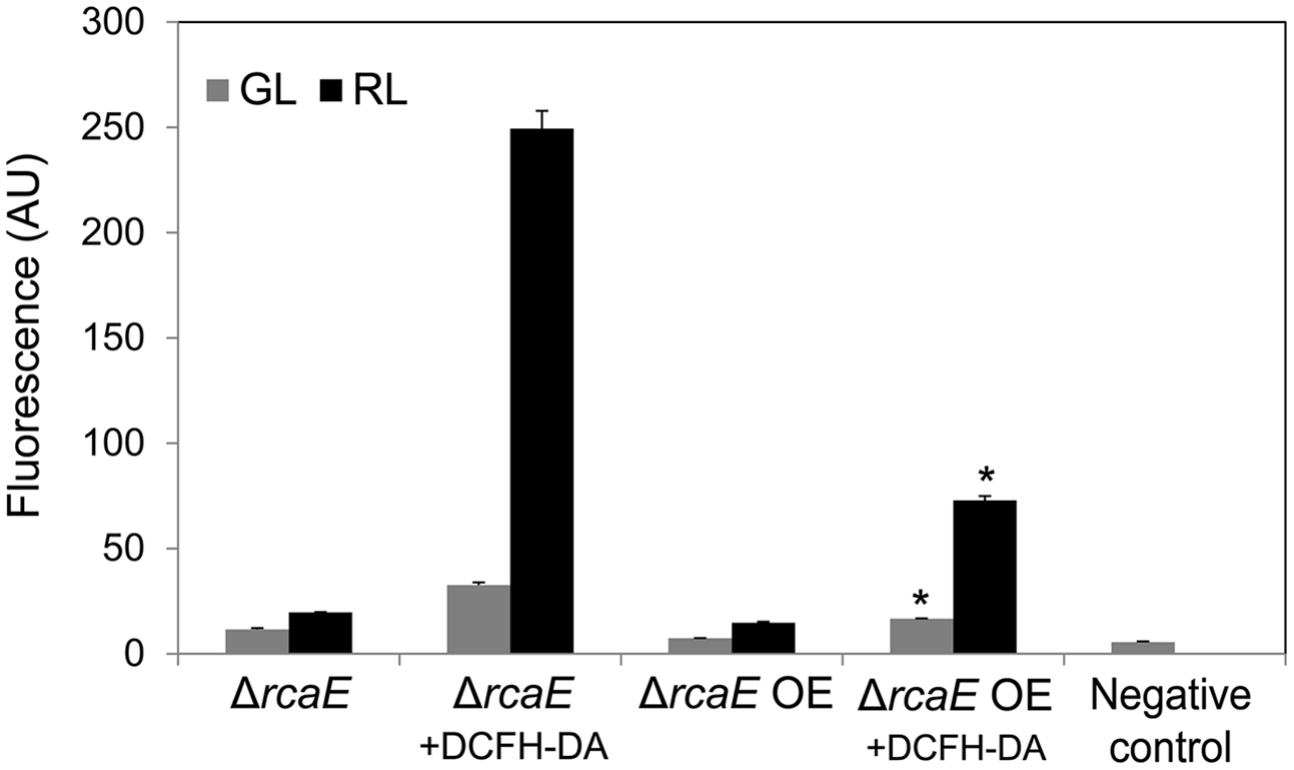
**Reactive oxygen species accumulation in *F. diplosiphon* Δ*rcaE* and Δ*rcaE* strain overexpressing *bolA* (Δ*rcaE* OE) grown under GL or RL.** Cellular component-associated fluorescence at 520 nm or ROS-dependent DCF fluorescence at 520 nm in arbitrary units (AU) after 72 h of growth of cells with DCFH-DA dye added (+DCFH-DA) under GL or RL. The negative control represents fluorescence originating from BG-11 medium + ROS-sensitive dye DCFH-DA without cells added. Asterisks indicate significant difference (*P* < 0.05) from Δ*rcaE* counterpart grown under GL or RL.

Similar to the WT OE strain, a higher accumulation of BolA in Δ*rcaE* OE was associated with decreased levels of ROS under GL and RL compared to Δ*rcaE* (**Figure 8**). In contrast to the observed impact on ROS levels, there was no noted difference in the growth response or whole-cell absorption spectra of Δ*rcaE* and Δ*rcaE* OE strains when grown under identical conditions (**Supplemental Figure S5**). However, the concentrations of PE, PC and AP were higher in Δ*rcaE* OE than in Δ*rcaE* under identical growth conditions (**Supplemental Figure S6B**). The concentration of chl *a* in Δ*rcaE* and Δ*rcaE* OE strains was similar under GL; however, the observed concentration of chl *a* in Δ*rcaE* OE was higher under RL than in Δ*rcaE* (**Supplemental Figure S6A**).

### Differences in Morphology in Distinct *F. diplosiphon* Strains Correlated with BolA Accumulation

**FIGURE 9:**
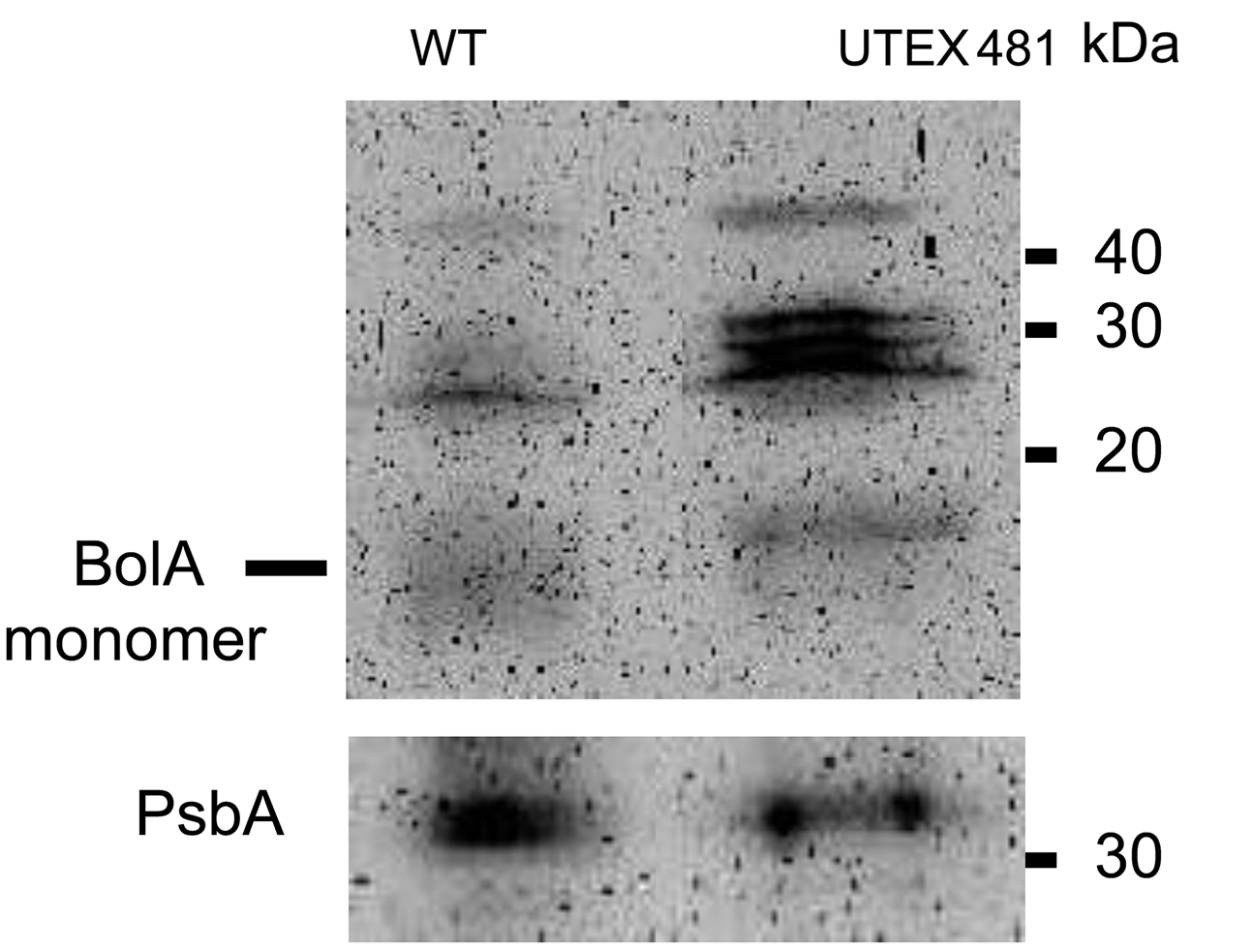
**Immunoblot analyses of BolA **(upper)** and PsbA **(lower)** accumulation in SF33 (WT) and UTEX 481 strains of *F. diplosiphon* grown under GL.** 150 μg of total protein extract from different strains of *F. diplosiphon* were separated on 15% SDS-PAGE. After blotting, BolA was detected using affinity-purified anti-BolA antibodies. After stripping of the blot to remove anti-BolA antibodies, the blot was probed with anti-PsbA antibodies to detect PsbA as a control of total protein quantification to account for differences in cellular volume and morphology of the two strains. A molecular mass in kilodalton (kDa) is indicated to the right.

*Fremyella diplosiphon* UTEX 481 strain, which is the parent of the shortened filament WT-pigmentation SF33 strain, is characterized by longer filaments than SF33 (Bordowitz and Montgomery, 2008). The longer UTEX 481 filaments are comparable to the longer filaments we observed in the WT OE strain (**Figure 3**). This observation suggested that longer filaments in UTEX 481 could be associated with its ability to accumulate higher cellular levels of BolA than the SF33 strain. To test this hypothesis, SF33 and UTEX 481 cultures were grown under identical GL conditions, and immunoblot analysis conducted to compare the levels of BolA in these two strains. UTEX 481 was found to accumulate higher levels of BolA in comparison to the SF33 strain (**Figure 9**). The relative abundance of PsbA was similar between these strains, suggesting the higher accumulation of BolA may be relevant to the observed differences in cellular volume and morphology between the two strains. UTEX481 also accumulated larger immunoreactive bands, similar to WT OE (compare **Figures 2A** and **9**).

## Discussion

The expression of morphogene *bolA* was found to be differentially regulated under GL and RL in a RcaE-dependent manner in *F. diplosiphon* (Singh and Montgomery, 2014). Furthermore, the light-regulated expression of *bolA* under GL and RL was inversely correlated with expression of *mreB* and *mreC* genes which are known to encode rod shape determining bacterial cytoskeleton proteins (Kruse et al., 2005; Cabeen and Jacobs-Wagner, 2007; Divakaruni et al., 2007; Singh and Montgomery, 2014). Based on results obtained from gene expression analyses, protein-DNA binding assays and functional characterization of *F. diplosiphon bolA* and *mreB* genes, we proposed a working model where RcaE promotes accumulation of BolA under RL, which leads to binding of BolA to the promoter of *mreB* and associated downregulation of *mreB* expression that is associated with RL-specific spherical morphology (Singh and Montgomery, 2014). Under GL, lower levels of BolA was predicted to promote MreB accumulation and GL-associated rod shaped cells (Singh and Montgomery, 2014). Here, we examined BolA protein levels under GL and RL in WT, Δ*bolA* and Δ*rcaE* strains and in strains with BolA overexpression to probe the relationship between BolA abundance and specific effects on cellular morphology regulation. Higher accumulation of BolA due to overexpression of *bolA* resulted in induction of RL-characteristic cellular morphology under GL in the WT OE strain. This observation strongly supports BolA accumulation as a critical factor for inducing spherical morphology due to RcaE-dependent higher expression of *bolA* under RL (Singh and Montgomery, 2014). Notably, the maximum permissible concentration of kanamycin for WT OE cultures was 3.5 μg/ml in contrast to a permissible concentration of 50 μg/ml for WT transformed with an empty vector. Higher concentrations were associated with lysis of WT OE cells evident by release of PBPs into the growth medium at higher kanamycin levels (data not shown). An inability to grow the WT OE strain at higher concentrations of kanamycin, i.e., above 3.5 μg/ml, could be associated with a prior correlation of increased antibiotic selection with maintenance of a higher cellular plasmid load and associated higher protein expression from plasmid-encoded genes (Chong et al., 2003; Bangen et al., 2004). This observation suggests that *F. diplosiphon* can tolerate only certain intracellular levels of BolA, and that higher accumulation of BolA beyond permissible levels is lethal for the cells. We have noted similar responses for other proteins in *F. diplosiphon* (Agostoni et al., 2015). By comparison, the Δ*rcaE* OE strain was able to grow initially at a concentration of 22.5 μg/ml kanamycin; however, cell lysis was evident in cultures maintained at higher concentrations of kanamycin after a few rounds of subculturing. Δ*rcaE* OE could be sustainably maintained at 6 μg/ml of kanamycin. Survival of Δ*rcaE* OE at a higher selection pressure compared to the WT OE strain, i.e., 6 μg/ml vs. 3.5 μg/ml of kanamycin, respectively, may be associated with the ability of Δ*rcaE* OE to support a higher level of BolA accumulation relative to its parental stain due to the lower initial endogenous level of BolA in Δ*rcaE* than in the WT strain (**Figure 1B**).

The requirement of functional RcaE for normal expression of *bolA* under GL and RL was clearly demonstrated by the fact that Δ*rcaE* strain accumulates lower levels of *bolA* transcript (Singh and Montgomery, 2014) and BolA protein (**Figure 1B**) than WT. In comparison to WT, Δ*rcaE* null mutant cells are characterized by large, round shape and high ROS levels (Bordowitz and Montgomery, 2008; Singh and Montgomery, 2012). The depletion of all but a few copies of *bolA* in Δ*bolA* strain, which results in low levels of BolA accumulation (**Figure 1**), was found to cause larger cell shape and increased levels of ROS relative to WT (Singh and Montgomery, 2014). Together, these findings suggested that morphological defects and high ROS levels in Δ*rcaE* cells could be due, at least in part, to aberration in expression of *bolA* and associated BolA accumulation in the absence of RcaE. Here, we show that higher accumulation of BolA in Δ*rcaE* OE results in decreased levels of ROS relative to the parent Δ*rcaE* and altered cellular morphology which was comparable to WT (**Figures 6** and **8**). This observation suggests that RcaE may function to repress ROS levels through BolA and that RcaE-dependent photoregulation of morphology may involve maintaining suitable cellular levels of BolA under GL and RL in *F. diplosiphon*.

The function of BolA as a reductase was put forward based on comparative genomic, phylogenetic distribution, and 3D structure analyses, and it was proposed that BolA can catalyze the reduction of ROS by obtaining reducing power from monothiol glutaredoxin (Huynen et al., 2005). Recently, interaction of BolA with monothiol glutaredoxin was established in yeast and *Arabidopsis thaliana*, which supports the proposition that BolA might obtain reducing power from monothiol glutaredoxin to catalyze the reduction of ROS (Huynen et al., 2005; Li et al., 2011; Couturier et al., 2014; Roret et al., 2014). The co-occurrence of the genes encoding monothiol glutaredoxin and BolA is highly conserved in eukaryotic and prokaryotic organisms (Huynen et al., 2005; Stroher and Millar, 2012). This conservation of co-occurrence of genes encoding BolA and monothiol glutaredoxin was also found in all but a few cyanobacteria, including *F. diplosiphon* (Singh and Montgomery, 2014). Reduced accumulation of BolA in the Δ*bolA* strain of *F. diplosiphon* resulted in increased levels of ROS under GL and RL, which is in accordance with proposed involvement of BolA in controlling intracellular ROS levels (Huynen et al., 2005; Singh and Montgomery, 2014). Overexpression of *bolA* results in decreased levels of ROS under GL and RL (**Figures 4** and **8**). These results further support involvement of BolA in the regulation of intracellular ROS (Huynen et al., 2005; Singh and Montgomery, 2014).

In addition to affecting cellular morphology and ROS levels of WT OE and Δ*rcaE* OE strains, increased levels of BolA accumulation in these strains was surprisingly associated with an unprecedented increase in the length of filaments (**Figures 3** and **7**). The observed increased filament length could be the result of decreased levels of ROS associated with higher accumulation of BolA in WT OE and Δ*rcaE* OE strains. Filament length of *F. diplosiphon* UTEX 481 was found to be increased in the presence of an antioxidant which reduces levels of ROS under GL and RL (Singh and Montgomery, 2012). Additionally, ROS accumulation has been shown to be associated with increased level of lipid peroxidation in cyanobacterial systems which could eventually impact integrity of filaments (He and Häder, 2002; Rastogi et al., 2010). RL-dependent higher accumulation of ROS in comparison to GL is associated with shorter filaments in WT *F. diplosiphon*, and it has been proposed that selective lysis of cells by oxidative stress results in fragmentation of filaments under RL (Bennett and Bogorad, 1973; Singh and Montgomery, 2012). UV-radiation dependent increased levels of ROS in cyanobacterial systems have been shown to be associated with fragmentation of filaments (Ma and Gao, 2009; Rastogi et al., 2010; Singh et al., 2014). However, direct involvement of BolA in maintaining intact filaments cannot be ruled out.

In summary, the present study supports a role for RcaE-dependent, morphogene-mediated regulation of cellular morphogenesis during CCA. We also provide evidence that the observed morphological and oxidative stress defects in the Δ*rcaE* strain could be associated with low levels of BolA. Results from our study also provide evidence that apposite regulation of intracellular ROS levels impacts filament length.

## Author Contributions

SS and BM conceived and designed experiments. SS conducted experiments. SS and BM analyzed data and wrote and edited the paper.

## Conflict of Interest Statement

The authors declare that the research was conducted in the absence of any commercial or financial relationships that could be construed as a potential conflict of interest.
