# Supplementary material for: Regulation of BolA abundance mediates morphogenesis in Fremyella diplosiphon
Source: Front Microbiol. 2015 Nov 5;6:1215. doi: 10.3389/fmicb.2015.01215 (PMC4633512; doi:10.3389/fmicb.2015.01215)
Supplement: Supplementary file 1 [file Supplemental_File.DOCX]

Supplementary Material

Regulation of BolA abundance mediates morphogenesis in *Fremyella diplosiphon*

Shailendra P. Singh, Beronda L. Montgomery^*^

*** Correspondence:** Corresponding Author: montg133@msu.edu

**Supplemental Table S1. Primers used in this study**

| **Primer name** | **Forward primer/FP (5’-3’)** | **Reverse primer/RP (5’-3’)** | **Purpose** |
| --- | --- | --- | --- |
| apcAp | CGTAAATCTGAAAAGTAATATTACAAAATAAGAGCAG | CTGCGGACTAATCATTAATGGATTCCTCCA | Overexpression construct |
| bolA | TGGAGGAATCCATTAATGATTAGTCCGCAG | CTAAGAAGCGGCTGTTGTCTG | Overexpression construct |
| bolART | TAGTCCGCAGCAGGTTGAGGAAAT | TGCCGCAAAGCACCGTAAACTAAC | qRT-PCR |
| mreBRT | TTTCGCTCATCGTGGGATATGGGT | CAAAGGACGGAGCGCAATCACATT | qRT-PCR |
| mreCRT | TAGTTGGACGTAGCGCTGACCATT | TGACGCTTTCTACCAAACCGACCA | qRT-PCR |
| mreDRT | ACTGGTCGCCGTCCAAATAAGTCA | GCGGGCTGGTAACAATAGCAAACA | qRT-PCR |
| orf10BRT | AGAACTACAGCGTCAGCTTAAT | CTGCTTCGCTTTCAGCATTT | qRT-PCR |

**Supplemental Table S2. Plasmids or constructs used in this study**

| **Construct** | **Selection Genotype^a^** | **Reference** |
| --- | --- | --- |
| pPL2.7GW | Cm^R^ Kan^R^ | ([Bordowitz & Montgomery, 2008](#_ENREF_1)) |
| pPL2.7 | Kan^R^ | ([Chiang *et al.*, 1992](#_ENREF_2)) |
| PCR^TM^8/GW/TOPO^®^ | Spn^R^ | (Invitrogen) |
| PCR^TM^8/GW/TOPO-*apcA*_p_*bolA* | Spn^R^ | This work |
| pPL2.7GW-*apcA*_p_*bolA* | Kan^R^ | This work |

^a^ Cm^R^, Km^R^, and Spn^R^ represent resistance to chloramphenicol, kanamycin and spectinomycin, respectively.


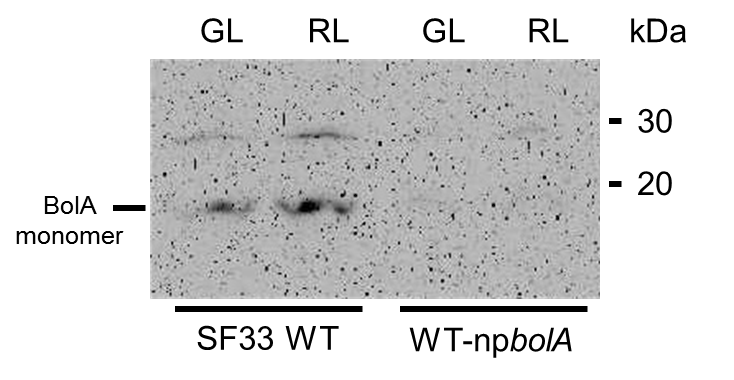


**Supplemental Figure S1**. Immunoblot analysis of BolA accumulation in *F. diplosiphon* wild-type (WT) and WT strain expressing *bolA* gene under the control of its endogenous promoter (WT-np*bolA*) under green light (GL) or red light (RL). 150 µg of total protein extract from different strains of *F. diplosiphon* were separated on 15% SDS-PAGE. After blotting, BolA was detected using affinity-purified anti-BolA antibodies. Molecular mass in kilodalton (kDa) is indicated to the right.


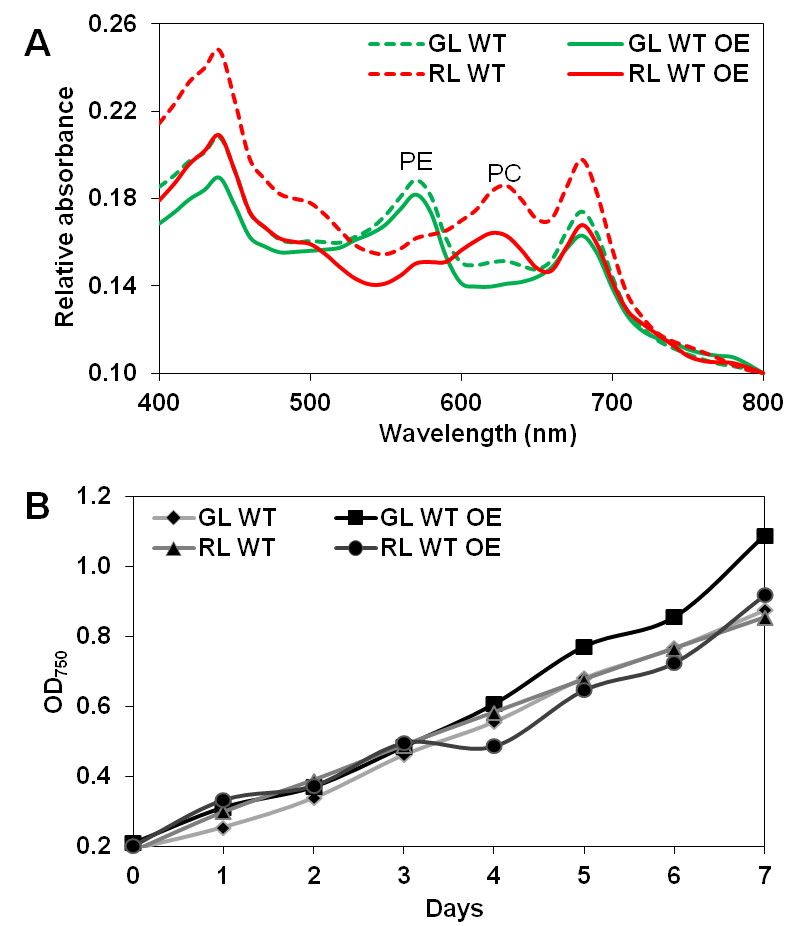


**Supplemental Figure S2**. Whole-cell absorption spectra and growth of *F. diplosiphon* WT and WT strain overexpressing *bolA* under the control of *apcA* promoter (WT OE) grown under green light (GL) or red light (RL). (A) WT and WT OE cultures were grown under GL or RL for 7 days, and spectral scans were taken between 400 – 800 nm after adjusting the optical density at 800 nm (OD_800_) to ~0.1. The peaks at 565 nm and 620 nm indicate accumulation of phycoerythrin (PE) and phycocyanin (PC), respectively. The outermost peaks are chlorophyll. (B) Growth of WT and WT OE strains under GL and RL measured every 24 h for consecutive 7 days as scattering of light at 750 nm (optical density or OD_750_).


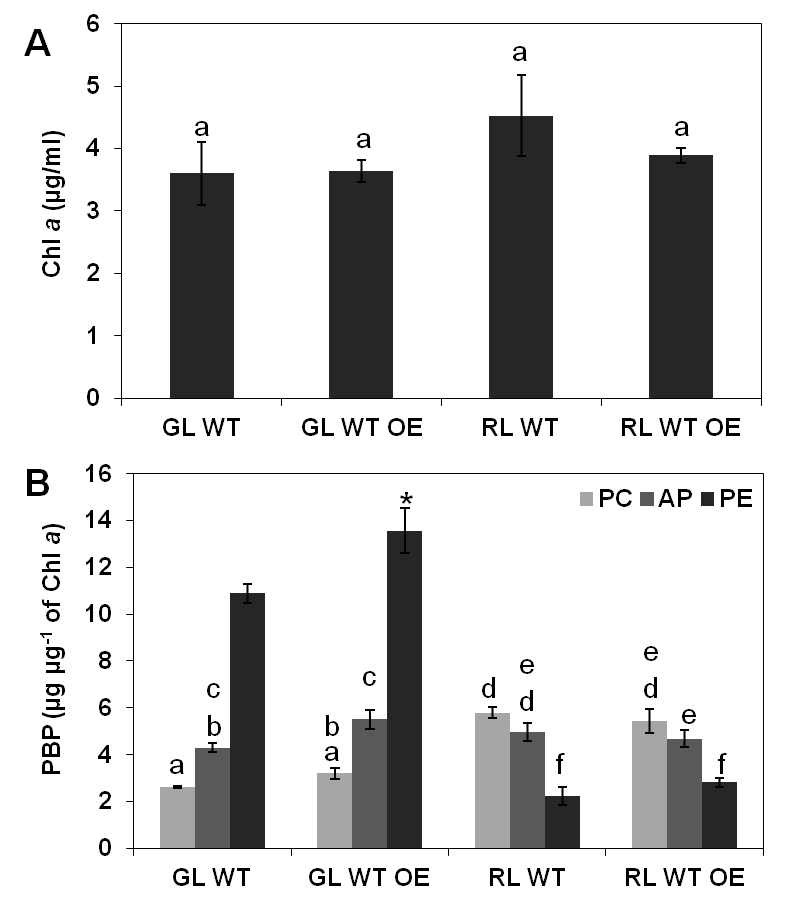


**Supplemental Figure S3**. Chlorophyll *a* (chl *a*) and phycobiliprotein contents of *F. diplosiphon* WT and WT strain overexpressing *bolA* under the control of *apcA* promoter (WT OE) grown under green light (GL) or red light (RL). (A) The concentration of chl *a* and (B) concentrations of phycoerythrin (PE), phycocyanin (PC) and allophycocyanin (AP) in WT and WT OE strains of *F. diplosiphon* after 7 days of growth under GL and RL. Asterisk indicates significant difference (p < 0.05) from WT for an indicated pigment, whereas similar letters over bars represent no significant difference (p > 0.05).


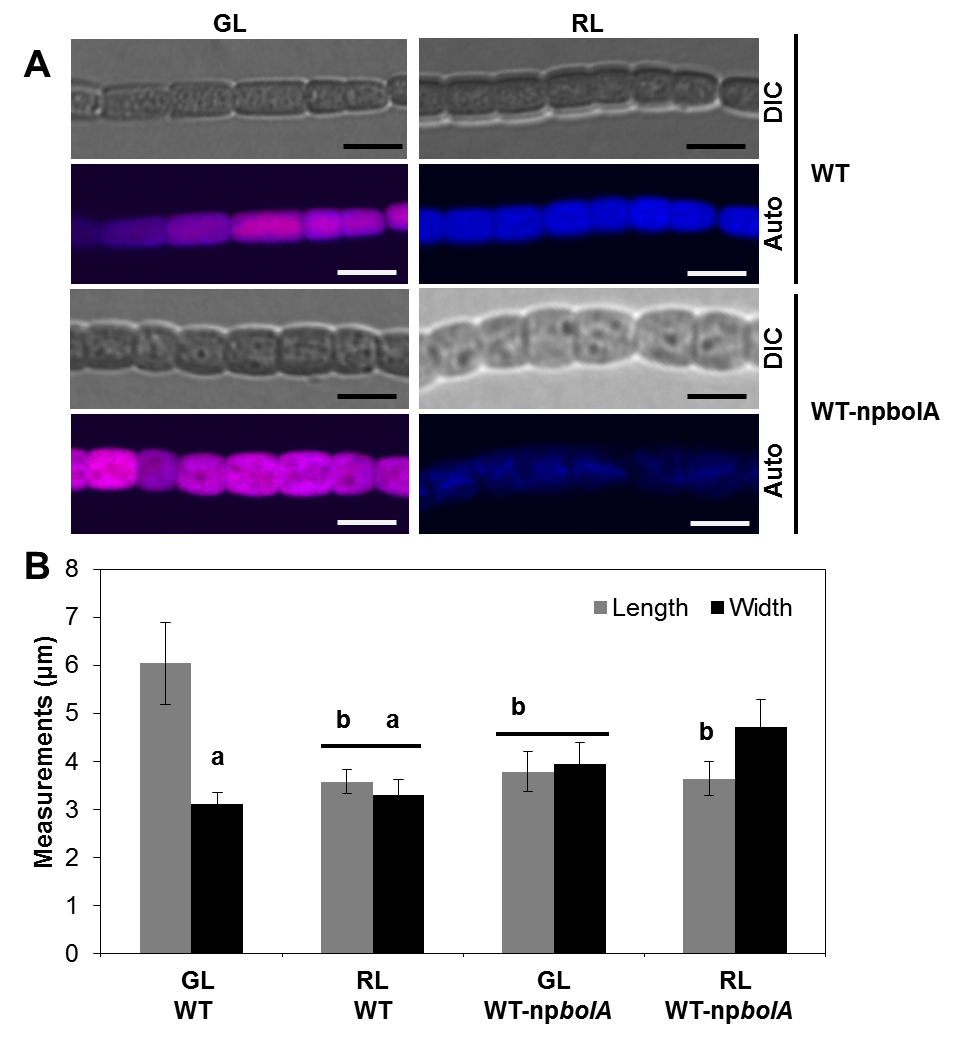


**Supplemental Figure S4**. Confocal laser scanning microscopy analyses of the cellular morphology of *F. diplosiphon* WT and WT strain overexpressing *bolA* under the control of its endogenous promoter (WT-np*bolA*) grown under green light (GL) or red light (RL). (A) Representative optical slices from a Z-series of differential interference contrast (DIC) images and corresponding maximum intensity projection PBP autofluorescence (auto) images of WT and WT OE strains grown under GL or RL for 72 h. Images were acquired using a 40× oil immersion objective with 2× zoom setting. Bars, 5 μm. (B) Cell length and width measurements of *F. diplosiphon* WT and WT OE strains grown under GL or RL for 72 h. Identical letters over bars represent a homogenous mean group (*P* > 0.05), whereas line over bars indicate no significant difference between the length and width of cells (*P* > 0.05) for a particular condition. No symbol over the bar indicates a significant difference (*P* < 0.05) from others.


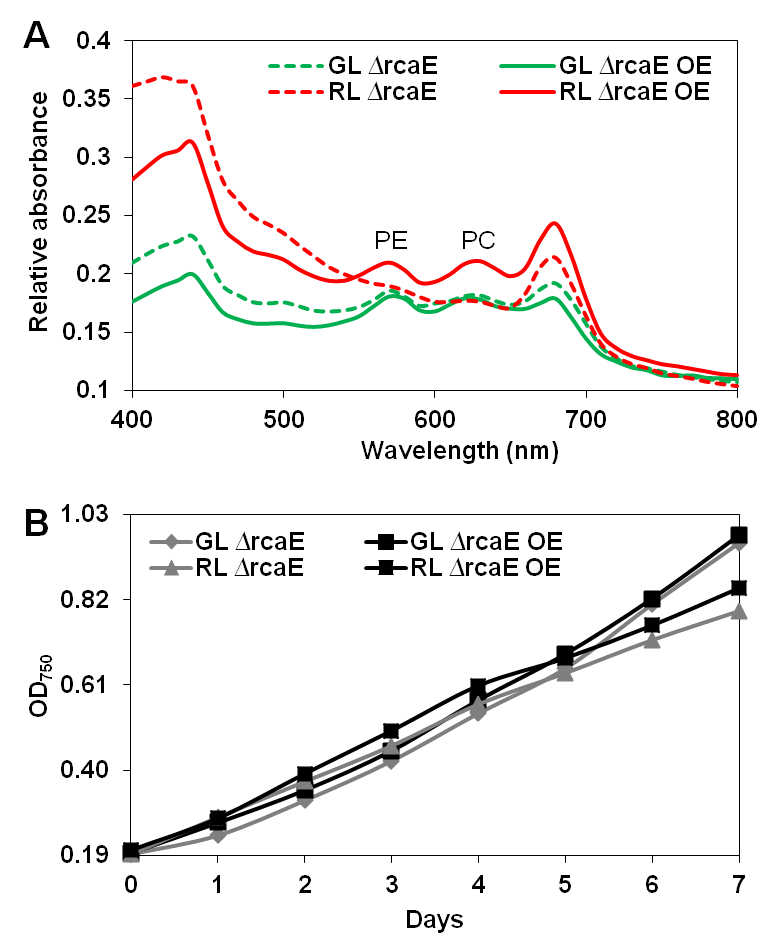


**Supplemental Figure S5**. Whole-cell absorption spectra and growth of *F. diplosiphon* ∆*rcaE* and ∆*rcaE* strain overexpressing *bolA* (∆*rcaE* OE) grown under green light (GL) or red light (RL). (A) ∆*rcaE* and ∆*rcaE* OE cultures were grown under GL or RL for 7 days, and spectral scans were taken between 400 – 800 nm after adjusting the optical density at 800 nm (OD_800_) to ~0.1. The peaks at 565 nm and 620 nm indicate accumulation of phycoerythrin (PE) and phycocyanin (PC), respectively. The outermost peaks are chlorophyll. (B) Growth of ∆*rcaE* and ∆*rcaE* OE strains under GL and RL measured every 24 h for consecutive 7 days as scattering of light at 750 nm (optical density or OD_750_).


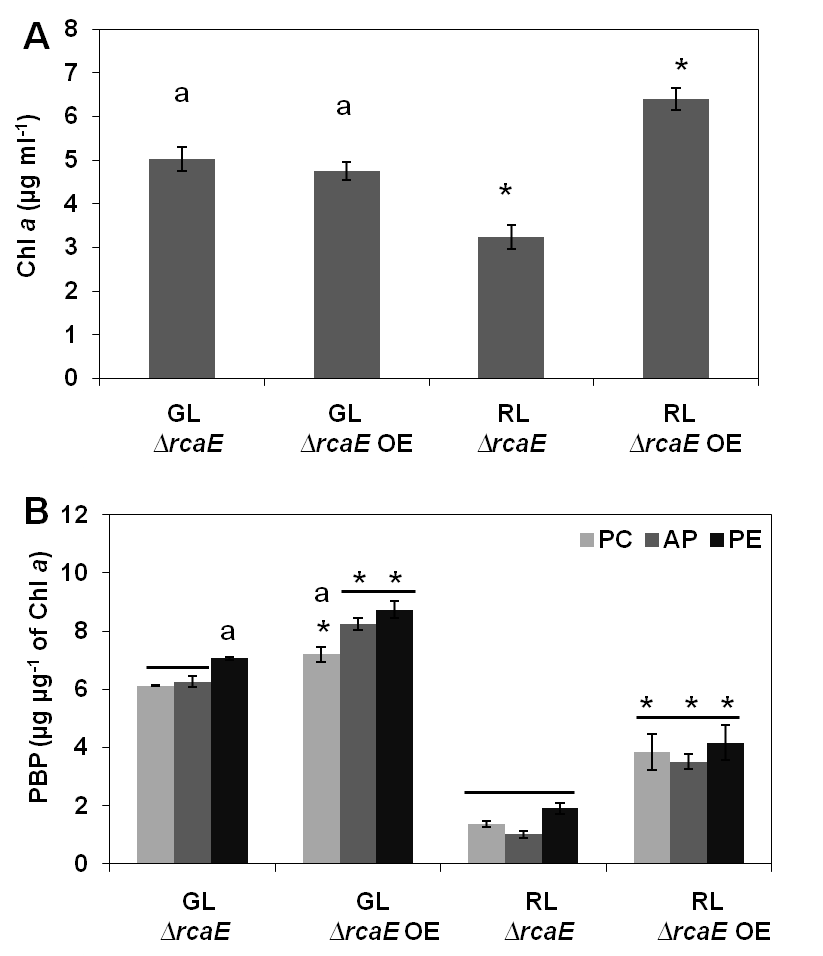


**Supplemental Figure S6**. Chlorophyll *a* (chl *a*) and phycobiliprotein contents of *F. diplosiphon* ∆*rcaE* and ∆*rcaE* strain overexpressing *bolA* (∆*rcaE* OE) grown under green light (GL) or red light (RL). (A) The concentration of chl *a* in ∆*rcaE* and ∆*rcaE* OE strains of *F. diplosiphon* after 7 days of growth under GL or RL. Asterisk indicates significant difference (p < 0.05) from others, whereas similar letters over bars represent no significant difference (p > 0.05). (B) The concentrations of phycoerythrin (PE), phycocyanin (PC) and allophycocyanin (AP) in ∆*rcaE* and ∆*rcaE* OE strains of *F. diplosiphon* after 7 days of growth under GL or RL. Asterisk indicates significant difference (p < 0.05) from ∆*rcaE* for an indicated pigment, whereas similar letters over bars represent no significant difference (p > 0.05). Line over bars indicate no significant (p > 0.05) difference between phycobiliproteins (PBP).

**References**

Bordowitz, J.R. and Montgomery, B.L. (2008) Photoregulation of cellular morphology during complementary chromatic adaptation requires sensor-kinase-class protein RcaE in *Fremyella diplosiphon*. *Journal of Bacteriology* **190**: 4069-4074.

Chiang, G.G., Schaefer, M.R. and Grossman, A.R. (1992) Transformation of the filamentous cyanobacterium *Fremyella diplosiphon* by conjugaison or electroporation. *Plant Physiology and Biochemistry* **30**: 315-325.
